# PARPs and PARP inhibitors: molecular mechanisms and clinical applications

**DOI:** 10.1186/s43556-025-00385-1

**Published:** 2025-12-29

**Authors:** Fei Wang, Zhuyi Guo, Michael J. Carr, Weifeng Shi

**Affiliations:** 1 Ruijin Hospital, Shanghai Jiao Tong University School of Medicine, Shanghai, China; 2 Shanghai Institute of Virology, Shanghai Jiao Tong University School of Medicine, Shanghai, China; 3 National Virus Reference Laboratory, School of Medicine, University College Dublin, Dublin, D04 E1W1 Ireland; 4 International Collaboration Unit, International Institute for Zoonosis Control, Hokkaido University, Sapporo, 001-0020 Japan; 5 School of Life Sciences and Biotechnology, Shanghai Jiao Tong University, Shanghai, China

**Keywords:** PARPs, PARPi, Cancer, Virus, Clinical applications

## Abstract

Poly (ADP-ribose) polymerases (PARPs) are a diverse family of enzymes that regulate genome stability, cell death, and stress responses through ADP-ribosylation. Among them, PARP1, PARP2, and PARP3 are central to cellular DNA repair, while tankyrases, and their isoforms, contribute to telomere maintenance, transcriptional regulation, immune signaling, and metabolism. Dysregulated PARP activity drives genomic instability, apoptosis, parthanatos, and tumor microenvironment remodeling, thereby linking PARPs to oncogenesis, immune escape, and therapy resistance. Clinically, PARP inhibitors (PARPi), such as olaparib, niraparib, rucaparib, and talazoparib, exploit synthetic lethality in homologous recombination–deficient tumors and are increasingly applied in ovarian, breast, prostate, and pancreatic cancers. Beyond oncology, preclinical studies demonstrate antiviral efficacy of PARPi against hepatitis B virus, human immunodeficiency virus, and coronaviruses, and also therapeutic potential in neurodegeneration, cardiovascular disease, fibrosis, and metabolic disorders. However, PARPi resistance arises through restoration of DNA repair, replication fork protection, epigenetic changes, and drug-target dynamics, while adverse events—including hematologic toxicity, gastrointestinal disturbance, and organ-specific effects—limit a broader use. Next-generation PARPi with improved isoform selectivity, PROteolysis-TArgeting Chimera (PROTAC) degraders, and rational combinations with ATR/CHK1 inhibitors, immune checkpoint blockade, or epigenetic modulators offer strategies to enhance efficacy and overcome resistance. Emerging biomarker-driven approaches, including liquid biopsies and functional assays, may further personalize therapy. By integrating canonical DNA repair roles with non-canonical signaling and host–virus interactions, PARPs represent pivotal regulators. Similarly, the versatile therapeutics of PARPi have implications that extend beyond oncology into a broader and diverse range of other human diseases.

## Introduction

Poly(ADP-ribose) polymerases (PARPs) are a family of enzymes that play essential roles in diverse cellular processes. Their initial discovery dates to 1963, when Chambon and colleagues identified a nuclear enzyme capable of synthesizing poly(ADP-ribose) (PAR) from nicotinamide adenine dinucleotide (NAD^+^), providing the first evidence of ADP-ribosylation as a novel post-translational modification [1]. Subsequent biochemical studies in the 1970s established PARP1 as the major nuclear isoform, which was rapidly recruited and activated in response to DNA double-strand breaks (DSBs) proving fundamental to DNA repair [2]. The cloning of PARP1 in the 1980s revealed its modular structure—including zinc-finger DNA-binding motifs, an automodification region, and a catalytic domain—which served to elucidate the mechanistic basis for its role in genome monitoring [3, 4]. A major conceptual advance emerged in the 1990s when genetic and pharmacologic studies demonstrated that PARP1 is indispensable for the base excision repair (BER) pathway, firmly linking PARP activity to both genome integrity and cellular survival [5]. Among the 17 PARP family members, PARP1 remains the most extensively studied enzyme and is central to cellular responses to DNA damage [6, 7]. The PARP family of enzymes, via poly-ADP ribosylation (PARylation), coordinate a diverse network of signaling pathways controlling genomic stability, transcriptional regulation, chromatin remodeling, and cell fate decisions [8].

The early 2000s marked a transformative era for the understanding of the PARP family of proteins with the emergence of the paradigm of synthetic lethality [9]. Pioneering studies in 2005 revealed that concurrent loss of both PARP1 activity and BRCA1/2-mediated homologous recombination (HR) resulted in catastrophic DNA damage and selective tumor cell death, which have fundamentally reshaped strategies for targeted cancer therapies. These insights directly enabled the development of the PARP inhibitors (PARPi), which block repair of single-strand breaks (SSBs), forcing their conversion into lethal DSBs in HR-deficient cancers [10, 11]. These mechanistic foundations supported the approval of multiple PARPi for an expanding range of malignancies, establishing PARPi as a new pillar of precision oncology [9]. Subsequent breakthroughs further accelerated the field, including the discovery of PARP-trapping as a distinct cytotoxic mechanism [12], and high-resolution structural studies of PARP1-DNA-inhibitor complexes via crystallography and cryo-electron microscopy, which revealed dynamic conformations essential for sensing DNA breaks [13, 14]. The subsequent identification of tankyrases (PARP5A/B) expanded PARP biology into telomere maintenance and Wnt signaling [15]. The discovery of catalytically inactive members, such as PARP9 and PARP13, together with mono-ADP-ribosylating enzymes, like PARP15, expanded the functional landscape of the PARP family into immune regulation and RNA metabolism, illustrating the plethora of cellular processes impinged upon and the evolving therapeutic scope for their clinical applications [16].

Despite these noteworthy advances, major challenges still remain, including tissue-specific toxicities, adaptive resistance, and an incomplete understanding of isoform-specific functions of PARPi. Today, the understanding of PARP biology has evolved from a single DNA repair enzyme to a multifunctional signaling network that governs genome maintenance, immunity, and metabolism [17, 18]. The development of next-generation PARPi, PROteolysis-TArgeting Chimera (PROTAC)-based PARP degraders, and isoform-specific inhibitors marks the latest phase in this trajectory, offering new opportunities to overcome resistance and extend therapeutic applications beyond oncology [19–21]. 

This review aims to integrate the current knowledge of PARP biology with clinical experience regarding PARPi, highlighting their expanding roles not only in malignancy but also in infectious and other non-malignant diseases. We begin by outlining the classification, structure, and multifaceted biological functions of PARPs—covering their canonical roles in DNA repair, apoptosis, and parthanatos, as well as their non-canonical functions in immunity, transcription, and metabolism. We then explore the pathologic mechanisms of action of PARPs in cancer, viral infections [22–25], and other diseases such as neurodegenerative, cardiovascular, and metabolic disorders [26–30]. Next, we detail the mechanisms of action of PARPi in both normal and malignant cells, followed by a comprehensive discussion of their clinical applications in oncology, antiviral therapy, and other disease settings. Finally, we address emerging challenges including drug resistance and safety concerns, and conclude with future perspectives on next-generation inhibitors and novel therapeutic strategies. By systematically organizing these themes, this review provides an overarching and up-to-date resource for researchers and clinicians interested in the broadening therapeutic landscape of PARP targeting to treat a diverse array of human diseases.

## Mechanisms and biological functions of PARPs

### Classification and structure of PARPs

**Fig. 1:**
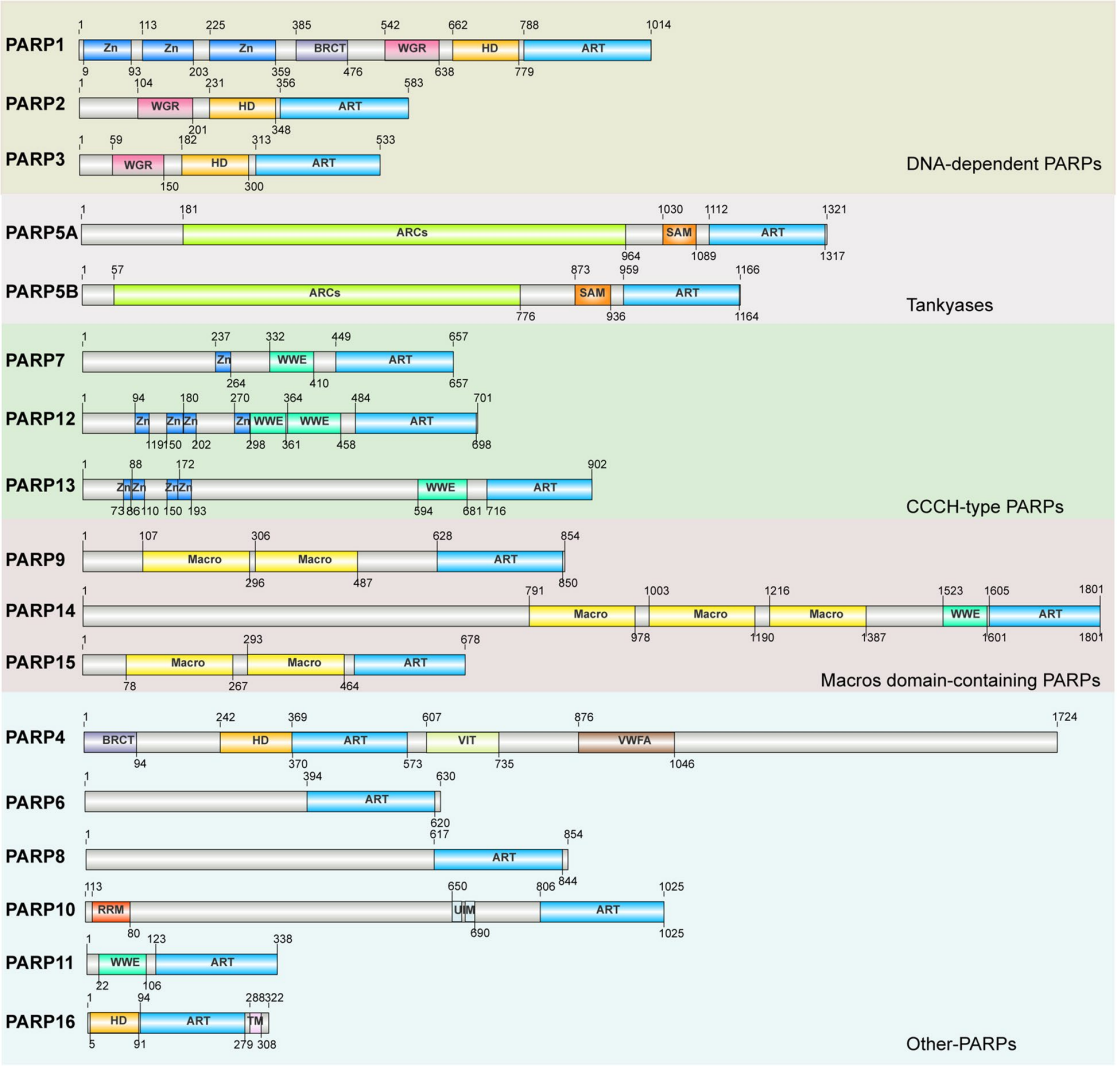
Domain organization and classification of human PARP family members. The figure is drawn based on the structures available from Uniprot. The 17 human poly(ADP-ribose) polymerases (PARPs) are grouped into five subfamilies based on their structural domains and functional features: DNA-dependent PARPs (PARP1, PARP2, and PARP3), Tankyrases (PARP5A, and PARP5B), CCCH-type PARPs (PARP7, PARP12, and PARP13), Macro domain-containing PARPs (PARP9, PARP14, and PARP15), and other PARPs with diverse structures (PARP4, PARP6, PARP8, PARP10, PARP11, and PARP16). ART (ADP-ribosyl transferase domain), essential for catalytic activity; Zn (Zn finger domain) and BRCT (BRCA C-terminal domain), involved in DNA binding and protein interactions; WGR (Trp-Gly-Arg domain), important for DNA-dependent activity; ARCs (ankyrin repeat cluster) and SAM (sterile alpha motif domain), characteristic of tankyrases for scaffolding functions; Macros domains, involved in ADP-ribose binding; HD (helical domain), important for enzymatic allosteric regulation and activation; VWFA (von Willebrand factor type A domain), mediating metal ion-dependent adhesion to partner proteins; WWE (Trp-Trp-Glu), VIT (vault protein interacting) domain and UIM (Ubiquitin Interacting Motif), mediating protein–protein interactions; RRM (RNA Recognition Motif), mediating the recognition and binding to damaged DNA

The PARP family of proteins comprise 17 enzymes that share a conserved catalytic core but differ markedly in their regulatory domains and biological functions (Fig. 1). Among these, PARP1, PARP2, and PARP3 are the most thoroughly characterized, largely due to their central roles in DNA repair and cellular stress responses [31]. PARP1, in particular, is rapidly activated upon sensing DSBs, catalyzing PARylation and coordinating the assembly of DNA repair factors necessary for safeguarding genome integrity [32, 33]. By contrast, PARP5A and PARP5B possess distinctive ankyrin-repeat clusters and sterile alpha motif (SAM) domains that mediate their functions in both telomere maintenance and the regulation of Wnt/β-catenin signaling [15, 34]. Other PARP family members—including PARP7, PARP12, and PARP13—participate primarily in innate immune responses, antiviral defenses, and transcriptional regulation [35]. Meanwhile, PARP9, PARP14, and PARP15, though lacking full enzymatic activity, operate as mono-ADP-ribosyltransferases or ADP-ribose-binding effectors that modulate diverse intracellular signaling pathways [16, 36].

Despite their functional diversity, the vast majority of PARPs contain a C-terminal catalytic domain defined by the conserved HYE (His-Tyr-Glu) tripeptide motif (Fig. 1), which catalyzes the transfer of ADP-ribose from NAD⁺ to target proteins [37]. This reversible post-translational modification plays a critical role in modulating the function of targeted proteins, chromatin organization, and intracellular signaling dynamics [37, 38]. In contrast, the N-terminal and accessory domains vary substantially across the PARP family members and largely dictate their functional specialization. PARP1, for example, contains three zinc-finger motifs that allow rapid recognition of DNA lesions, an automodification region that regulates chromatin accessibility, and a WGR (Trp-Gly-Arg) domain that mediates DNA binding and intramolecular communication [14, 39]. PARP2 and PARP3 retain the WGR domain but lack zinc-finger modules, corresponding to their more focused involvement in SSB repair and chromatin association [14, 38]. The PARP5A/B enzymes feature extended ankyrin-repeat arrays that mediate protein interactions and drive substrate specificity, as well as SAM domains that promote polymerization and amplify downstream signaling events [40]. Catalytically attenuated members, such as PARP9, PARP13, and PARP15, instead employ macrodomains, RNA-binding motifs, or WWE (Trp-Trp-Glu) modules to regulate immune signaling and RNA metabolism [16, 41, 42]. Together, the conserved catalytic architecture paired with highly diversified regulatory domains underpins the diverse functional spectrum of the PARP family (Fig. 1).

### PARPs in cellular homeostasis

#### The role of PARPs in DNA repair

PARPs are activated in response to SSBs or DSBs in DNA repair, particularly PARP1, PARP2, and PARP3, which are primary responders to cellular DNA damage. They recognize DNA SSBs or DSBs via their structural domains and rapidly recruit repair machinery by catalyzing the PARylation or mono(ADP-ribosyl)ation (MARylation) of substrate proteins. This activity initiates and coordinates various repair pathways, including BER, single-strand break repair (SSBR), and double-strand break repair (DSBR) [43, 44]. As the most extensively studied member, PARP1 is involved in HR and non-homologous end joining (NHEJ), highlighting its multifaceted role in maintaining genome integrity [45–47]. The PAR strand synthesized by PARP1 also promotes chromatin remodeling, allowing better access of repair proteins to damaged DNA [47]. Furthermore, this critical function is the foundation for the use of PARPi in cancer therapy, which exploit "synthetic lethality" to selectively target tumors with HR deficiency, representing a major breakthrough in targeted oncology [48].

#### The role of PARPs in transcriptional regulation and chromatin remodeling

Recent studies have substantially broadened the conceptual framework of PARP biology, positioning PARP1 and related family members as active modulators of transcription rather than passive accessory factors. PARP1 is now recognized as a chromatin-associated regulator that fine-tunes promoter accessibility and shapes RNA polymerase II (Pol II) behavior during early transcriptional events. Through PARylation of histones and pause-associated factors, PARP1 can influence the transition from Pol II pausing to productive elongation, thereby enabling rapid transcriptional responses to stress and developmental cues [49]. This regulatory activity operates in a locus-specific manner: PARP1 stabilizes chromatin compaction at metabolically hyperactive regions while promoting accessibility at poised promoters, effectively redistributing transcriptional output according to cellular demand [50].

Beyond PARP1, recent work highlights the involvement of mono-ADP-ribosyltransferases (mono-ARTs), such as PARP7 and PARP14, in remodeling transcription factor networks. PARP7 has been shown to MARylate aryl hydrocarbon receptor (AHR), altering their DNA-binding behavior and downstream transcriptional programs [51, 52], whereas PARP14—often functioning with PARP9-DTX3L complexes—helps integrate cytokine signaling with chromatin-embedded transcriptional states [53, 54]. An emerging theme across these findings is that ADP-ribosylation acts as a tunable regulatory layer that intersects with chromatin structure and Pol II–proximal complexes to influence transcriptional outputs [55]. Importantly, recent evidence suggests that PARP1 also senses DNA:RNA hybrids (R-loops) formed during transcription and coordinates their resolution with RNase H2 and chromatin remodelers, positioning PARP activity at a critical interface between transcription and genome stability [56]. Notably, new findings further demonstrate that PARP1-dependent resolution of R-loops requires the coordinated action of EXD2 and CBP: when PARP1 is depleted or its catalytic activity is inhibited, EXD2 and CBP fail to localize to R-loop sites, leading to impaired hybrid removal [57]. Collectively, these discoveries support a shift in perspective: PARPs should be viewed not only as DNA-repair enzymes but as dynamic regulators of transcriptional circuitry whose activity influences both gene expression patterns and cellular homeostasis.

#### The role of PARPs in apoptosis

Beyond their role in DNA repair, PARPs are also involved in the regulation of apoptosis. In the early stages of apoptosis, caspase-3 is activated and cleaves PARP1, splitting it into two segments: a 24 kDa N-terminal and an 89 kDa C-terminal fragment [58]. This cleavage event is considered one of the quintessential hallmarks of apoptosis as it causes PARP1 to lose its DNA repair function and thereby promote cell death [59]. At the same time, excessive activation of PARP1 is also closely related to apoptosis in some viral infections. For example, certain DNA and RNA viruses trigger apoptosis by inducing DNA damage or by activating PARP1, thereby promoting virus release and transmission [60, 61]. In addition, PARP1 also triggers apoptosis by interacting with the stimulator of interferon genes (STING) protein [62]. Conversely, certain members like the tankyrases (PARP5A/B) [63], PARP9 [64], and PARP14 [65], exhibit anti-apoptotic functions, promoting cell survival. This dual and often opposing role in apoptosis and parthanatos highlights the complex and critical regulatory network governed by the PARP family in determining cell fate, whose dysregulation is intimately linked to tumorigenesis and therapy resistance.

#### The role of PARPs in parthanatos

Parthanatos is a distinct form of regulated cell death primarily mediated by PARP1. It is characterized by a series of biochemical events that lead to cell demise without the activation of caspases, which are the hallmark of apoptosis [66]. Upon sensing DNA damage, PARP1 becomes hyperactivated, leading to the rapid synthesis of PAR chains that modify various nuclear proteins. This process is crucial for the release and nuclear translocation of apoptosis-inducing factor (AIF), where it interacts with macrophage migration inhibitory factor (MIF) to enhance its nuclease activity, leading to extensive DNA fragmentation [67, 68]. Moreover, the depletion of NAD^+^ and ATP due to PARP1 activation also contributes to cellular energy failure and promotes parthanatos [69]. The interplay between PARP1 activity and cellular energy status is vital for determining cell fate, making PARP1 a promising therapeutic target in diseases where parthanatos is implicated. For example, inhibition of PARP1 has been shown to alleviate neuronal damage in models of neurodegenerative diseases and ischemic injury, highlighting the importance of this pathway in therapeutic interventions [70–72].

#### Non-canonical functions of PARPs

Beyond canonical DNA repair and apoptosis, many PARP members play significant roles in a broad spectrum of cellular processes (Table 1). PARP1 participates in various biological processes such as ferroptosis [73], necroptosis [74, 75], pyroptosis [76], cellular metabolism [113], transcriptional regulation, and the cell stress response [114], thereby contributing to both immunomodulation and cellular invasiveness. PARP5A and PARP5B are involved in regulating cell proliferation through telomere maintenance and the Wnt/β-catenin signaling pathway [48, 63]. PARP7 [48], PARP13 [108], and PARP14 [54] act as critical regulators of innate immune responses, modulating immune microenvironments via interferon signaling. These non-canonical functions highlight the expansive influence of the PARP family proteins in cellular signaling networks and suggest novel perspectives for clinical trials involving combinatorial therapeutic strategies. It is important to note that not all PARP family members possess catalytic activity, e.g. PARP9 and PARP13, yet they still play key roles as scaffold proteins, molecular adaptors, or signal modulators via their protein-interaction domains (macrodomains and zinc fingers). For instance, PARP9 and PARP13 have been reported to facilitate the assembly of signaling complexes or function as RNA-binding proteins to regulate gene expression [115]. Even among the catalytically-active members, their protein products vary: PARP1, PARP2, PARP4, PARP5A, and PARP5B synthesize long-chain PAR polymers [77, 116], while most other members primarily catalyze MARylation [77]. This diversity in catalytic activity and molecular functions adds a significant layer of complexity to the regulatory repertoire of the PARP family.

**Table 1:** Classification of the PARP family members with key domains and functions

PARP Member	Functions				Enzymatic Activity	Reference
	**DNA Repair**	**Apoptosis**	**Parthanatos**	**Others**		
PARP1	SSBR, DSBR	Pro-apoptotic under severe DNA damage	Primary inducer via PAR accumulation	Ferroptosis, necroptosis, pyroptosis, mitosis, cell cycle, metastasis, transcription, chromatin structure modulation, membrane repair and cell stress response	PAR, MAR	[6, 48, 73–83]
PARP2	SSBR, DSBR	May suppress apoptosis	/	Mitosis, chromatin structure modulation, telomere maintenance	PAR	[6, 48, 82, 84–89]
PARP3	SSBR, DSBR	/	/	Mitosis, centrosome regulation, chromatin structure modulation, transcription	PAR, MAR	[6, 48, 77, 80, 82, 84, 86, 90, 91]
PARP4	DSBR	Pro-apoptotic	/	Cell transportation and vault particle regulation	PAR, MAR	[48, 92–94]
PARP5A (Tankyrase-1)	DSBR	Anti-apoptotic	/	Pyroptosis, cellular metabolism, telomere maintenance, mitosis, cell division, necroptosis	PAR	[6, 48, 63, 95–97]
PARP5B (Tankyrase-2)	DSBR	Anti-apoptotic	/	Pyroptosis, mitosis, cellular metabolism, telomeric and centromeric regulation	PAR	[48, 63, 97]
PARP6	/	/	/	Cell structure, adhesion, motility, spindle pole regulation and cell replication	MAR	[48, 98]
PARP7	/	Anti-apoptotic	/	Transcription regulation, cell structure, adhesion and motility, innate immunity, and cell stress response	MAR	[48, 99–101]
PARP8	/	/	/	Membrane and nuclear envelope formation	MAR	[48]
PARP9	DSBR	Anti-apoptotic	/	Lipid metabolism, innate immunity	Catalytically Inactive	[64, 77, 80, 102–104]
PARP10	Nascent strand DNA gaps repair, translesion synthesis (TLS), DSBR	Pro-apoptotic	/	Lipid metabolism, spindle pole regulation and cell replication	MAR	[36, 48, 102, 105, 106]
PARP11	/	/	/	Spermatogenesis, membrane and nuclear envelope formation	MAR	[48]
PARP12	/	Anti-apoptotic	/	Necroptosis, mouse oocyte meiotic maturation, cell stress response	MAR	[48, 107]
PARP13	/	Pro-apoptotic in viral infection	/	Innate immunity	Inactive	[48, 77, 80, 108, 109]
PARP14	Homologous Recombination (HR), Replication Fork Protection, SSBR	Anti-apoptotic	/	Lipid metabolism, transcription regulation, cell structure, adhesion, motility and innate immunity	MAR	[36, 48, 65, 102]
PARP15	/	/	/	Post-translational modification of proteins and negative regulator of transcription	MAR	[48]
PARP16	/	Pro-apoptotic	/	ER stress response, cell stress response, membrane and stabilization of amyloid precursor protein mRNA	MAR	[48, 110–112]

*BER* base excision repair, *SSR* single-strand break, *ER* Endoplasmic reticulum, *MAR* MARylation, *PAR* PARylation

## PARPs in disease pathogenesis

### Mechanisms of PARPs in cancers

#### PARPs and genomic instability

PARPs collectively maintain genomic stability through distinct yet complementary mechanisms, and their dysregulation serves as a critical driver of tumorigenesis. PARP1 acts as a central sensor and catalytic effector by recognizing SSBs and catalyzing PAR chains to recruit DNA repair factors, primarily governing BER [117, 118]. PARP1 dysfunction leads to the accumulation of SSBs that can progress into lethal DSBs, directly promoting genomic instability. PARP2 shares overlapping functions with PARP1, playing essential roles in SSBR and DSBR, thereby contributing to genome integrity [119]. Compared with SSBR, PARP3 is more involved in DNA DSBR, influencing genomic stability through the regulation of HR and NHEJ [120]. Additionally, PARP5A and PARP5B contribute indirectly, though significantly, by regulating both telomere maintenance and modulating oncogenic signaling pathways. In cases of telomere dysfunction, DSBs can be triggered, affecting chromosome stability [48, 63]. Moreover, various other PARP family members are activated by distinct forms of DNA damage, and serve to orchestrate cellular responses to this damage, thereby modulating genomic instability (Table 1). Therefore, the dysfunction of PARPs will aggravate genomic instability from multiple levels (DNA repair, replication, and chromosome maintenance), thus driving the occurrence and subsequent development of tumors.

#### The role of PARPs in tumor cell death

PARPs play a dual role in tumorigenesis by regulating multiple cell death pathways: on the one hand, members such as PARP2, PARP5A/B, PARP7, and PARP14 inhibit apoptosis and, thus, serve to promote cancer development. PARP2 enhances nasopharyngeal carcinoma growth via SIRT1/AMPK signaling [88]. PARP5A/B promotes PTEN degradation via PARylation to activate PI3K-AKT signaling and can also modulate AXIN stability activating WNT/β-catenin pathways, thereby inhibiting apoptosis [63, 121]. PARP7 ADP-ribosylates FRA1 to suppress IRF1/IRF3-dependent apoptosis [99] and, finally, PARP14 inhibits the pro-apoptotic gene JNK1 to support metabolic growth in hepatocellular carcinoma [65]. On the other hand, however, PARP16 inhibits tumor cell growth and metastasis by activating the endoplasmic reticulum stress signaling pathway and inducing apoptosis [110]. PARP4, stimulated by interferon gamma (IFN-γ) and tumor necrosis factor-alpha (TNF-α), mediates apoptosis in human neuroblastoma cells [92]. PARP1 also exerts context-dependent effects: it promotes apoptosis to eliminate DNA-damaged cells early in tumorigenesis, but excessive activation depletes NAD^+^ and ATP, triggering parthanatos and inhibiting cancer progression [122]. During radiotherapy, radiation-induced PAR polymers, synthesized by PARP1, bind to STING and induce apoptosis [123]. Conversely, inhibiting PARP1 suppresses tumor growth through two mechanisms: inducing ferroptosis by repressing SLC7A11, or triggering pyroptosis via caspase-3-mediated gasdermin E cleavage [73, 76]. Additionally, PARP1 variably modulates autophagy via the MRPL21-PARP1 axis to promote chemoresistance in head and neck squamous cell carcinoma [124], while being activated by UHRF2 to promote autophagy and malignancy in hepatocellular carcinoma [125]. In the HT-29 human colon adenocarcinoma cell line, T lymphocytes can sensitize IFN-γ-induced PARP cleavage and promote tumor cell apoptosis [126]. In oesophageal carcinoma cells, IFN-λ1 induces apoptosis by inducing the cleavage of PARP, thus inhibiting the growth of cancer cells [127]. These multifaceted mechanisms underscore the therapeutic potential of targeting PARPs in combinatorial therapeutic strategies, involving chemotherapy, radiotherapy, and immunotherapy which requires further investigation in clinical trials to assess additive and synergistic effects.

#### The role of PARPs in the regulation of the tumor microenvironment

PARPs are also increasingly acknowledged for their involvement in the modulation of the tumor microenvironment (TME), which affects both tumor development and the anti-tumor immune responses [128–130]. PARPs are actively engaged in the regulation of inflammatory responses within the TME, which may exacerbate tumor growth, contingent upon specific circumstances. For example, PARP7 promotes immune escape and tumor growth in the TME by negatively regulating the nucleic acid sensing-mediated type I interferon signaling pathway in tumor cells and inhibiting the inflammatory response [101]. PARP1 activates the IL-6-STAT3-cyclin D1 signaling axis to promote the progression of rectal cancer [131]. PARP14 can enhance the inflammatory response by supporting the polarization of macrophages to the tumor-promoting M2 type, and, thus, promote the occurrence and development of multiple tumors, such as breast cancer [132]. Conversely, PARP14 can also promote inflammatory cell infiltration by enhancing the expression of the IL-4-induced inflammatory cell chemokines CCL17 and CCL22, thus exacerbating the occurrence and development of follicular lymphoma [133]. Furthermore, PARPs regulate tumor cell migration and invasiveness by affecting microtubule stability. PARP7 is able to MARylate α-tubulin to promote microtubule instability, which may enhance the growth and motility of ovarian cancer cells [134]. The ability of PARPs to modulate the TME highlights their importance not only in tumor biology but also within the therapeutic arena, suggesting that targeting PARP activity may improve the efficacy of both chemo-, radio- and immunotherapies by reshaping the TME to support anti-tumor immunity [135–137]. The dynamic interaction between PARPs, immune response, and microtubule stability offers a complex framework for cancer treatment, indicating that approaches aimed at modulating PARP activity could potentially serve to further improve therapeutic efficacies [138, 139].

#### The role of PARPs in replication stress tolerance and fork protection

Replication stress is increasingly recognized as a defining vulnerability of many cancers, and recent studies have clarified how multiple PARP family members safeguard replication fork integrity under such conditions. PARP1 is recruited to stalled replication forks, where it helps prevent Mre11-mediated nascent DNA degradation and supports fork stability and restart under replication stress [140]. Emerging evidence suggests that PARP2 may also contribute to fork stabilization in specific contexts [141]. More recent work shows that PARP1 also facilitates fork reversal, enabling template switching when forks encounter obstacles, although dysregulated PARP1 activity can lead to pathological hyper-reversal, fueling genomic instability and tumor evolution [47, 142]. In addition, PARP10 interacts with PCNA via its PIP-box motif and recruits RAD18 to promote PCNA mono-/poly-ubiquitination, thereby facilitating translesion DNA synthesis (TLS) and enabling cells to bypass DNA lesions under replicative stress conditions [143]. Collectively, these findings indicate that PARPs function as a hub linking replication-fork remodeling to DNA repair, enabling cancer cells to survive chronic replication stress. Therapeutically, disrupting fork-protective functions of PARPs may offer a strategy to selectively exploit replication stress–associated weaknesses in tumors with oncogene-driven hyperproliferation.

#### The role of PARPs in cancer epigenetics and transcriptional reprogramming

Increasing evidence demonstrates that the influence of PARPs on tumor biology extends into the regulation of chromatin architecture and transcriptional programs. PARP1 remodels cancer-associated chromatin by PARylating the nucleosomal core histones and chromatin-bound regulatory factors, thereby promoting chromatin accessibility at promoters and supporting c-MYC- and E2F-driven proliferative gene expression in tumor contexts [144]. Furthermore, PARP7 fine-tunes AHR-dependent transcriptional outputs in lung and liver cancers, directly regulating the expression of oncogenic networks [145]. PARP14, frequently overexpressed in hematologic malignancies, reinforces IL-4/STAT6 transcriptional circuits, promoting tumor cell survival and metabolic adaptation [133, 146]. Studies highlight that PARP-mediated ADP-ribosylation functions as a versatile epigenetic modification that integrates metabolic state, immune signaling, and cytokine inputs to reprogram oncogenic transcription [147, 148]. These mechanistic insights underscore the potential of PARPs as master regulators that couple chromatin dynamics to tumor-promoting transcriptional networks, providing a strong therapeutic rationale for combining PARP inhibition with transcription- and/or chromatin-targeted therapies.

### Mechanisms of PARPs in virus infection

#### The role of PARPs in RNA virus infection

**Fig. 2:**
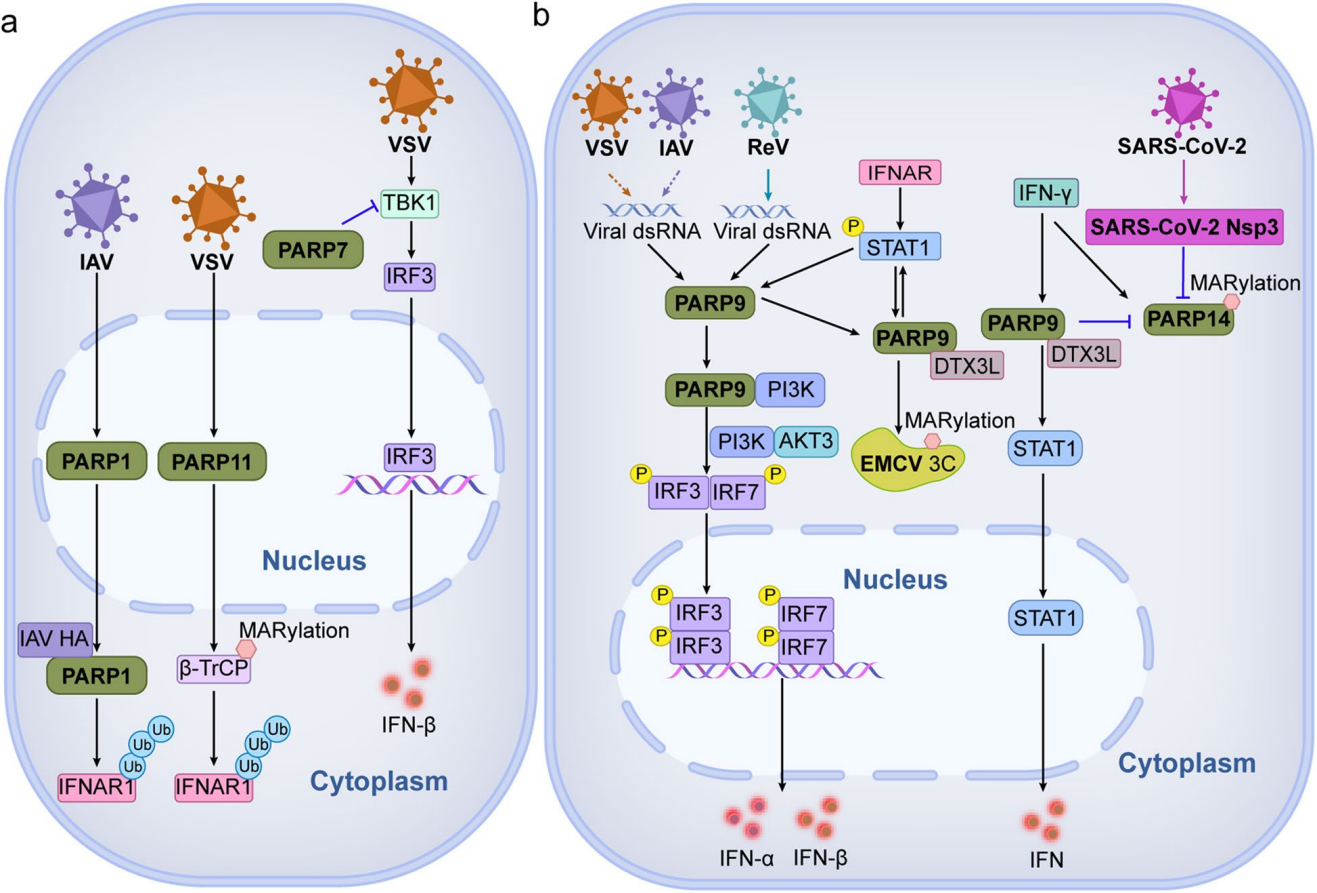
PARPs leverage the IFN signaling pathway to regulate host immunity upon RNA virus infection. **a** PARP1 and PARP11 promote IAV and VSV replication, respectively, by facilitating the ubiquitination of IFNAR. PARP7 promotes VSV replication by inhibiting the TBK1-IRF3 signaling pathway and reducing the production of IFNβ. **b** PARP9 can inhibit the replication of VSV, IAV, ReV and EMCV by promoting the production of IFN. In SARS-CoV-2 infection, IFN-γ-induced PARP14 can cooperate with PARP9 to regulate the production of IFN and inhibit viral replication

Crucially, PARPs can inhibit (Fig. 2a) or promote (Fig. 2b) the type I interferon (IFN) signaling pathway through multiple mechanisms during RNA virus infection, thereby affecting the host's antiviral immune responses. The influenza A virus (IAV) hemagglutinin (HA) protein can induce the degradation of the type I IFN receptor 1 (IFNAR1), and PARP1 plays a key role in this process. IAV infection induces cytoplasmic translocation of PARP1, and PARP1 interacts with HA to promote the ubiquitination and degradation of IFNAR1, thereby inhibiting the activation of the IFN signaling pathway (Fig. 2a) [24]. Upon vesicular stomatitis virus (VSV) infection, PARP11 is able to MARylate the ubiquitin E3 ligase β-TrCP, promoting its binding to IFNAR1, eventually resulting in ubiquitination and degradation of IFNAR1 (Fig. 2a). This process inhibits the activation of the IFN signaling pathway and attenuates host antiviral responses [149]. Meanwhile, PARP7 can inhibit TBK1 phosphorylation, thereby negatively regulating the production of IFN in VSV infection (Fig. 2a) [150].

In contrast, PARP9 specifically recognizes the dsRNA genome of Reovirus (ReV), initiating a signaling cascade that activates interferon regulatory factor 3 (IRF3) and interferon regulatory factor 7 (IRF7) through the PI3K/AKT3 signaling pathway, promoting IFN production. Importantly, this process does not rely on the classical mitochondrial antiviral signaling protein (MAVS) innate immune adaptor signaling pathway, but, instead, operates by enhancing IFN signaling by direct phosphorylation of key residues of IRF3 and IRF7. Although studies have not clearly determined whether PARP9 directly binds to dsRNA produced by IAV and vesicular stomatitis virus (VSV), infection can activate the same signaling pathway induced by PARP9, which in turn promotes the production of type I interferon. This suggests that PARP9 may activate the antiviral signaling pathway by directly binding to the dsRNA of IAV and VSV, or by other indirect mechanisms, in response to infection with these viruses which warrants further studies (Fig. 2b) [151]. PARP9-DTX3L, as an E3 ubiquitin ligase complex, can interact with STAT1 and enhance the transcriptional activity of STAT1, thereby promoting the expression of interferon-stimulated genes (ISGs). The PARP9-DTX3L complex is also able to directly target the encephalomyocarditis virus (EMCV) 3C protease, degrading these viral proteins through the ubiquitin–proteasome system (Fig. 2b), inhibiting viral replication [152]. Similarly, in SARS-CoV-2 infection, IFN-γ induces ADP-ribosylation by activating the PARP9/DTX3L complex and PARP14. PARP9 and PARP14 can synergistically inhibit viral replication. PARP9-DTX3L can remove the ADP modification catalyzed by PARP14 to regulate macrophage activation. The Nsp3 of SARS-CoV-2 can also hydrolyze this modification, thereby inhibiting the host's antiviral responses (Fig. 2b) [54, 153].

**Fig. 3:**
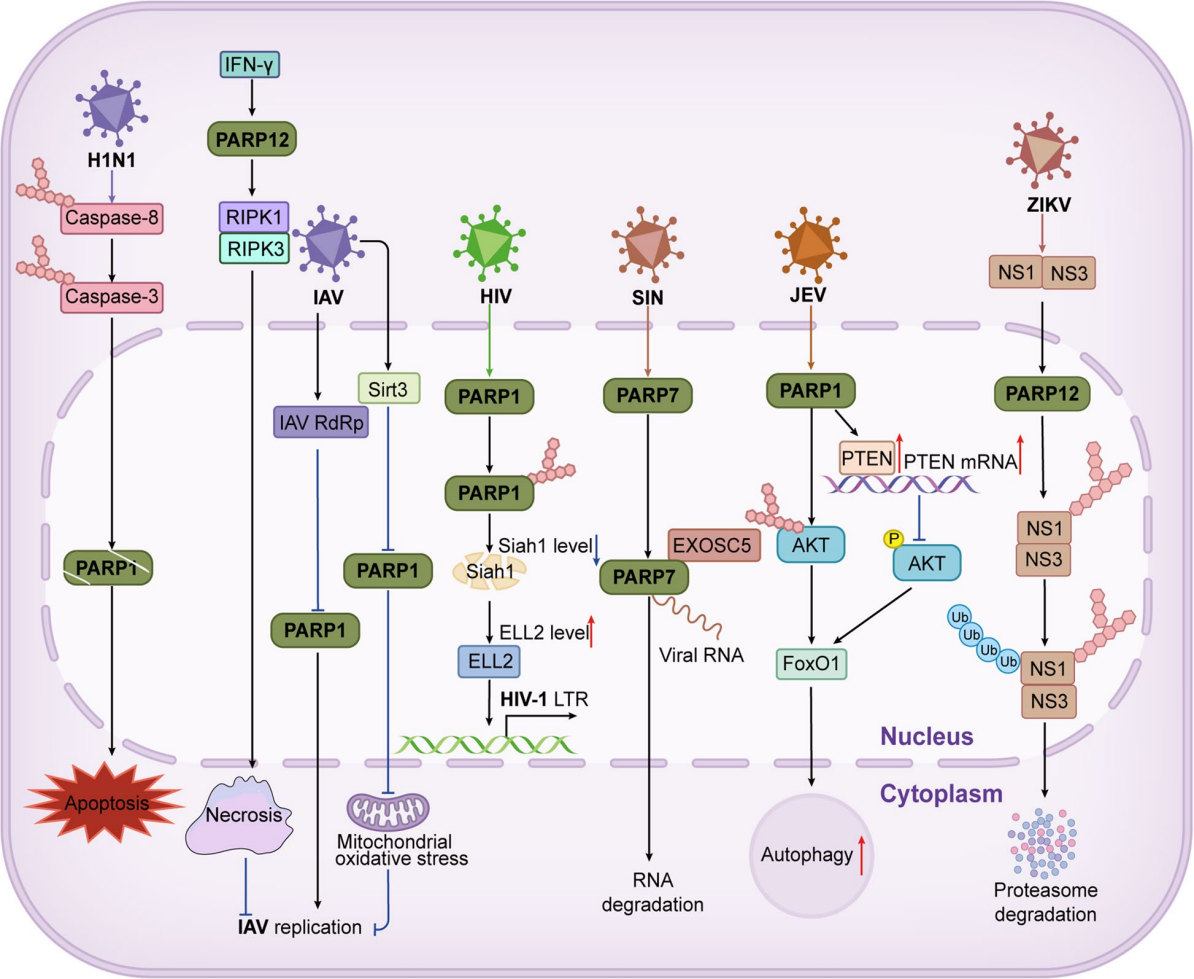
PARP modulates immune responses in response to RNA virus invasion through multiple pathways. PARP1 mediates apoptosis and regulates IAV replication. IFN-γ-induced PARP12 mediates necrosis and inhibits IAV replication. The role of PARP1 enzyme activity in IAV infection is complex and can be inhibited by RdRp to promote viral replication, and also Sirt3 inhibition can alleviate mitochondrial oxidative stress. PARP1 inhibits the expression of Siah1 and increases the level of the ELL2 protein, which in turn promotes the transcription of HIV. PARP1 activates transcription of PTEN and AKT, which in turn promotes autophagy to regulate JEV replication. PARP12 is able to ADP-ribosylate NS1 and NS3 proteins, resulting in their ubiquitination and proteasomal degradation, inhibiting ZIKV replication

In addition to modulating the IFN signaling pathway, PARPs can also regulate host immunity through pathways such as apoptosis, RNA degradation, viral transcription, interaction with host proteins, autophagy, and proteasome degradation (Fig. 3). IAV infection induces caspase-3 activation, which subsequently triggers PARP1 cleavage, culminating in apoptosis [154]. In IAV infection, IFN-γ-induced PARP12 regulates RIPK1 and RIPK3 through ADP-ribosylation and promotes RIPK1-RIPK3-mediated programmed cell necroptosis [155]. Furthermore, PARP1 can inhibit IAV replication following the activation of the PARP1 enzymatic activity. The inhibition of the PARP1 enzymatic activity can promote the RNA-dependent RNA polymerase (RdRp) activity of IAV and virus replication though the specific mechanisms are still unclear [156, 157]. Sirtuin 3 (Sirt3) decreases IAV replication and the associated inflammatory damage, oxidative stress, and mitochondrial dysfunction by inhibiting the PARP1 activity [158]. PARP7 can bind to Sindbis virus (SINV) RNA and exosome complex component 5 (EXOSC5) through its zinc finger domain and promote viral RNA degradation, thereby inhibiting SINV replication (Fig. 3) [159]. PARP1 promotes ELL2-super elongation complex (SEC) formation by increasing the level of ELL2 protein and inhibiting the expression of E3 ubiquitin ligase Siah1, which is crucial for the transcriptional activation of human immunodeficiency virus (HIV) [23].

PARPs play critical roles during the infection cycle of the important and genetically-related flaviviruses Japanese encephalitis virus (JEV) and Zika virus (ZIKV). During JEV infection, PARP1 can cause PARylation of AKT and affect its phosphorylation. At the same time, PTEN transcription can be upregulated, and then AKT is negatively regulated. PARP1-mediated AKT inactivation promotes autophagy and JEV pathogenesis (Fig. 3) [160]. Recently, studies have found that PARP12 is able to ADP-ribosylate the NS1 and NS3 proteins of ZIKV and can inhibit ZIKV replication (Fig. 3), leading to their ubiquitination and proteasomal degradation [161]. Collectively, these findings suggest that multiple PARPs respond differently to RNA virus infections with distinct antiviral strategies.

#### The role of PARPs in DNA virus infection

**Fig. 4:**
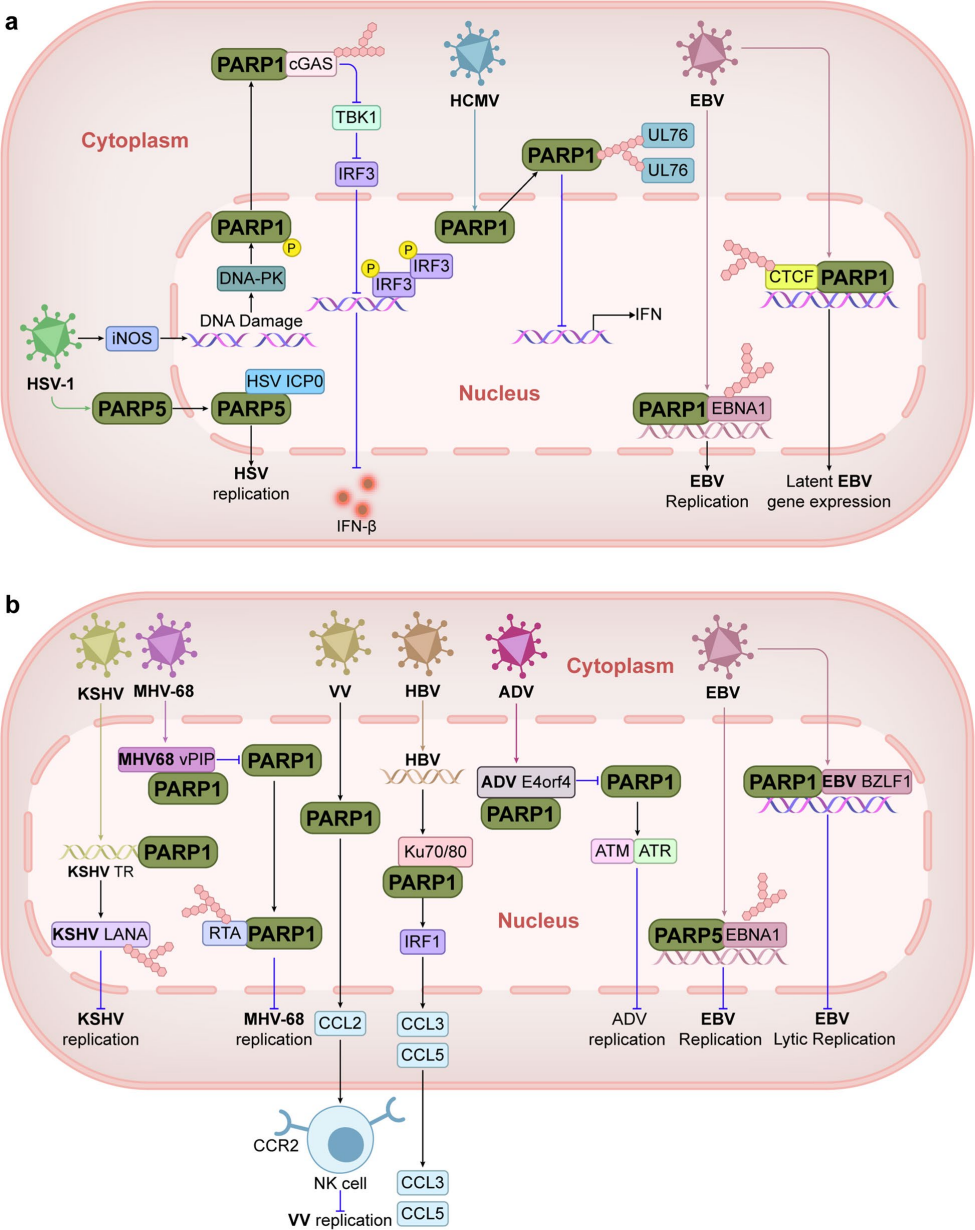
PARPs regulate immune responses to DNA viral infection via multiple cellular pathways. **a** PARP1 mediates the cGAS/STING signaling pathway through cytosolic translocation and promotes HSV-1 replication. PARP5 promotes HSV-1 replication through nuclear translocation. PARP1 binds to the UL76 protein of HCMV, inhibits IFN production, and promotes viral replication. PARP1 binds to EBNA1 and promotes replication and maintenance of the EBV genome. At the same time, PARP1 can also maintain the latent state of the EBV genome by stabilizing the CTCF binding site. **b** PARP1 modifies KSHV LANA and MHV68 RTA by PARylation to inhibit the replication of KSHV and MHV-68. PARP1 recruits NK cells through the CCL2-CCR2 axis, thereby inhibiting VV replication. PARP1 senses HBV DNA via the Ku70/80 complex, activates IRF1 and promotes secretion of CCL3 and CCL5 chemokines. PARP1 activates the ATM and ATR signaling pathways by sensing SSBs and DSBs, thereby inhibiting ADV replication. PARP5 binds to EBNA1 and inhibits EBV replication, and PARP1 is also able to bind to the BZLF1 promoter region of EBV, restricting EBV cleavage reactivation

Some DNA viruses can take advantage of the enzymatic activity of PARP or promote the protein modification activities of PARP to facilitate viral replication (Fig. 4a). Herpes simplex virus type 1 (HSV-1) infection activates the DNA-dependent protein kinase (DNA-PK), promoting PARP1 phosphorylation and nuclear-to-cytoplasmic transport, which modifies the cyclic guanosine monophosphate (GMP)–adenosine monophosphate (AMP) synthase (cGAS) protein through PARylation, inhibiting its DNA binding ability and diminishing IFN responses [162, 163]. Interestingly, HSV-1 infection induces PARP5A phosphorylation via ERK, thereby promoting its expression and translocation to the nucleus by interacting with ICP0, thus enhancing HSV replication [164]. During another herpesvirus infection, human cytomegalovirus (HCMV; human herpesvirus 5, HHV-5), PARP1 is also activated and transferred from the nucleus to the cytoplasm, promoting viral replication. The UL76 protein of HCMV binds to the BRCT domain of PARP1, maintaining the host cell survival [165]. In Epstein-Barr virus (EBV; human herpesvirus 4, HHV-4) infection, PARP1 may modify EBNA1 through PAR, promoting the replication and maintenance of the EBV genome. Recent studies have shown that PARP1 can maintain the three-dimensional structure and latent state of the EBV genome by stabilizing the CTCF binding site (Fig. 4a) [166, 167].

The role of PARPs in DNA viruses is therefore multifaceted, and PARP can also inhibit viral replication through alteration of enzymatic activities, protein modification, and recruitment of immune cells (Fig. 4b). PARP1 can directly bind to the terminal repeat (TR) sequences of Kaposi’s sarcoma-associated herpesvirus (KSHV; human herpesvirus 8, HHV-8) and modify the latency-associated nuclear antigen (LANA) protein through PARylation to inhibit the replication of KSHV during the incubation period. The activity of PARP1 affects both the stability and replication efficiency of the viral genome, and is a key factor in the maintenance of the KSHV incubation period [168]. PARP1 inhibits replication of murine gammaherpesvirus 68 (MHV-68) by PARylation of the replication and transcription activator (RTA) protein, a key switch molecule in the lytic replication of MHV-68. The vPIP protein encoded by MHV-68 interacts with PARP1 to prevent the inhibition of RTA by PARP-1, thereby releasing the inhibition of RTA by the PARylation event (Fig. 4b) [169].

In a model of intraperitoneal vaccinia virus (VV) infection, PARP1 recruits NK cells to the site of infection, through the CCL2-CCR2 axis, thereby facilitating viral control (Fig. 4b) [7]. PARP1 senses hepatitis B virus (HBV) DNA in the cytoplasm through the DNA repair protein Ku70/80 complex, activates IRF1 and promotes the secretion of CCL3 and CCL5 chemokines (Fig. 4b), thereby recruiting immune cells and triggering hepatitis [22]. PARP1 activates the ATM and ATR signaling pathways by sensing intermediate products, such as SSBs and DSBs, produced during adenovirus (ADV) DNA replication, thereby inhibiting ADV replication. ADV can bind to PARP1 through its E4orf4 protein and inhibits its activity reducing PARP1-mediated DNA damage signaling and promoting viral replication [170]. In EBV infection, PARP5 binds to EBNA1 and inhibits the replication of EBV (Fig. 4b) [171]. PARP1 is also capable of binding to the BZLF1 promoter region of EBV (Fig. 4b), restricting EBV lytic reactivation [172]. In conclusion, during DNA infection, PARPs regulate the antiviral responses by multiple pathways.

### Crosstalk mechanisms between cancer and viral infections

PARPs represent a critical node where cancer biology and viral infection intersect, as both contexts exploit DDR, metabolic reprogramming, and innate immune pathways. Mechanistically, cytoplasmic PARP1 can PARylate cGAS to curb its DNA binding, attenuate STING activation, and blunt type I interferon output—an immune-evasion strategy exploited during HSV-1 infection [163]. At the interface linking the DDR and innate immunity, PARP inhibition increases unresolved DNA and cytosolic DNA fragments, thereby activating cGAS–STING and enhancing antitumor immunity (and its synergy with checkpoint blockade), underscoring that the bidirectional control of immunity by PARPs is dependent on both context and timing [173, 174]. Metabolically, PARP activation consumes NAD^+^ and perturbs redox and energy flux. Live-cell imaging shows that PARP1-dependent NAD^+^ depletion drives a metabolic shift after DNA damage, illustrating how PARP signaling can reprogram bioenergetics in both stressed cancer and virus-infected cells [69]. Beyond PARP1, PARP14 directly promotes aerobic glycolysis (the Warburg effect) in hepatocellular carcinoma by modulating PKM2, linking ADP-ribosylation to pro-tumor glycolytic bias [175]. Viral infections likewise press on the same axis: coronaviruses induce MARylation of PARPs and deplete cellular NAD^+^, reconfiguring host metabolism and interferon responses in ways that can be countered by boosting NAD^+^ salvage [176]. Together, these data place PARPs at a shared mechanistic frontier of oncology and viral disease—coordinating DDR-derived innate sensing, immune evasion, and metabolic rewiring—and motivate the development of therapeutic strategies that co-target PARP/cGAS-STING and NAD^+^ metabolism in virus-associated cancers.

### Mechanisms of PARPs in other diseases

#### PARPs in neurodegenerative diseases, cardiovascular diseases and fibrosis

Members of the PARPs family are, via distinct mechanistic effects, intimately involved in a variety of pathological processes. In neurodegenerative diseases, overactivation of PARP1 mediates neuroinflammation and triggers energy-exhausting cell death, and abnormal PARylation accelerates toxic protein aggregation [177]. The upregulation of PARP2/3 plays a dual role between DNA repair and apoptosis risk [178]. PARP14 affects the inflammatory response by regulating the M1/M2 polarization of microglia [179]. PARP14 inhibitors can promote the polarization of microglia to the anti-inflammatory M2 type, thereby reducing inflammation and improving cognitive function in Alzheimer's disease [179]. In cardiovascular diseases, PARP1 is a core regulatory factor, which drives atherosclerosis and vascular remodeling by regulating the phenotypic transformation of vascular smooth muscle cells, and leads to myocardial hypertrophy and fibrosis by promoting inflammatory response [180, 181]. In fibrotic diseases, PARP5A and PARP5B promote pathogenic fibroblast activation and tissue remodeling by overactivation of the Wnt/β-catenin signaling pathway [182, 183], while PARP1 also promotes fibrosis by regulating inflammatory signaling pathways, such as HMGB1-NF-κB [184].

#### PARPs in inflammatory and autoimmune disorders

PARPs also play critical and diverse roles in inflammatory and autoimmune disorders through regulation of key immune processes. PARP1 promotes the production of inflammatory factors by regulating signaling pathways, such as NF-κB, while the activation of the NLRP3 inflammasome directly leads to the maturation and release of inflammatory factors, which together aggravates intestinal inflammatory damage [185]. PARP14 promotes Th2 cell differentiation and IgE production in inflammation-related diseases, such as asthma and allergy, by regulating Stat6-dependent gene activation, thereby promoting disease progression [186]. The proinflammatory cytokines TNF-α and IFN-γ exacerbate *Clostridioides difficile* infection (CDI)-triggered apoptosis in enteric glial cells (EGCs) by increasing caspase-3/7/9 and PARP activation [187]. In autoimmune diseases, the balance between Th17 and regulatory T (Treg) cells is critical for disease pathogenesis. PARP1 and PARP14 play important roles in modulating this balance. Studies have demonstrated that PARP1 activation promotes Th17 cell differentiation while simultaneously suppressing Treg cell generation [188]. In contrast, PARP14 activation enhances Treg cell function and inhibits Th17 cell activity [28]. This bidirectional regulatory mechanism underscores the importance of PARP family members in immune modulation. Additionally, in experimental autoimmune encephalomyelitis (EAE), PARP2 regulates the development of neuroinflammation and neurological dysfunction by affecting the infiltration of proinflammatory Th1 and Th17 T helper lymphocytes in the central nervous system [189]. Consequently, therapeutic strategies targeting PARPs may offer promising possibilities for restoring Th17/Treg homeostasis, thereby providing potential novel interventions for the management of autoimmune disorders.

#### PARPs in metabolic diseases

PARPs play key roles in metabolic diseases and their mechanism of action involves a plethora of effects. For example, in fatty acid metabolism, PARPs affect the metabolic processing of fatty acids by regulating the expression of genes related to fatty acid synthesis and oxidation. Changes in the enzymatic activities of PARP1 and PARP2 can lead to increased fatty acid synthesis or decreased oxidation, which in turn affects fat accumulation in adipose tissue and the liver [190, 191]. In terms of cholesterol and lipoprotein metabolism, PARPs regulate key processes in cholesterol synthesis, transport, and lipoprotein metabolism. Deletion or inhibition of PARP2 can lead to increased cholesterol synthesis [192], while inhibition of PARP1 can improve cholesterol transport and lipoprotein metabolism [193, 194]. In addition, PARPs also affect the progression of metabolic diseases by regulating pathways such as the inflammatory response, oxidative stress, and cellular energy metabolism. For example, in non-alcoholic fatty liver disease (NAFLD) and alcoholic fatty liver disease (AFLD), activation of PARP1 and PARP2 leads to increased fat accumulation in hepatocytes [195, 196], while PARP inhibition attenuates these pathological changes [190, 195]. In obesity and type 2 diabetes, PARPs affect insulin sensitivity and energy metabolism by regulating metabolic processes in adipose tissue and muscle. Activation of PARP1 can inhibit lipolysis in adipose tissue and fatty acid oxidation in muscle, thus exacerbating insulin resistance [190, 191]. In atherosclerosis, the activation of PARP1 can promote the infiltration of inflammatory cells and the formation of foam cells, exacerbating the progression of atherosclerosis [194, 197].

## Mechanisms of action of PARPi

### Therapeutic targets and pharmacological properties of PARPi

PARPi are a class of small-molecule agents rationally designed to interfere with the catalytic function of PARP enzymes, particularly PARP1 and PARP2, which play central roles in SSB repair. Structurally, most clinically developed PARPi mimic nicotinamide, the natural substrate of PARP, and competitively bind to the conserved nicotinamide-binding pocket within the catalytic domain. This binding prevents the transfer of ADP-ribose units from NAD^+^ to target proteins, thereby blocking the formation of PAR chains that are essential for recruiting DNA repair factors [198]. In addition to catalytic inhibition, many PARPi also exert a so-called “PARP-trapping” effect, whereby the drug stabilizes PARP-DNA complexes, physically obstructing DNA replication and transcription and creating cytotoxic lesions [9]. This dual mechanism of action—catalytic inhibition plus PARP trapping—underpins their ability to induce synthetic lethality in tumors with HRR deficiencies, such as those harboring BRCA1/2 mutations [199].

Several PARPi, such as olaparib, rucaparib, niraparib, talazoparib, pamiparib, fluzoparib, and senaparib, have been developed and approved for clinical use. While all share the common strategy of targeting the catalytic NAD^+^ binding site, they differ in binding affinity, PARP isoform selectivity, trapping potency, and pharmacokinetic properties. For instance, olaparib, the first FDA-approved agent in this class, is indicated for BRCA-mutated ovarian and breast carcinomas [200]. Niraparib is employed as maintenance therapy in recurrent ovarian cancer, irrespective of the BRCA mutational status, while rucaparib is effective in tumors with HR deficiency [201]. Talazoparib demonstrates superior PARP-trapping activity, whereas veliparib shows favorable synergy with DNA-damaging chemotherapy [202].

Recent generations of PARPi, such as fluzoparib [203], pamiparib [204], and senaparib [205], exhibit enhanced PARP1 selectivity, stronger antitumor efficacy, and reduced off-target effects. These advances have not only expanded the therapeutic landscape for hard-to-treat cancers but also refined the paradigm of precision oncology by enabling more durable and tolerable treatment options for patients.

### Mechanisms of PARPi in normal cells

**Fig. 5:**
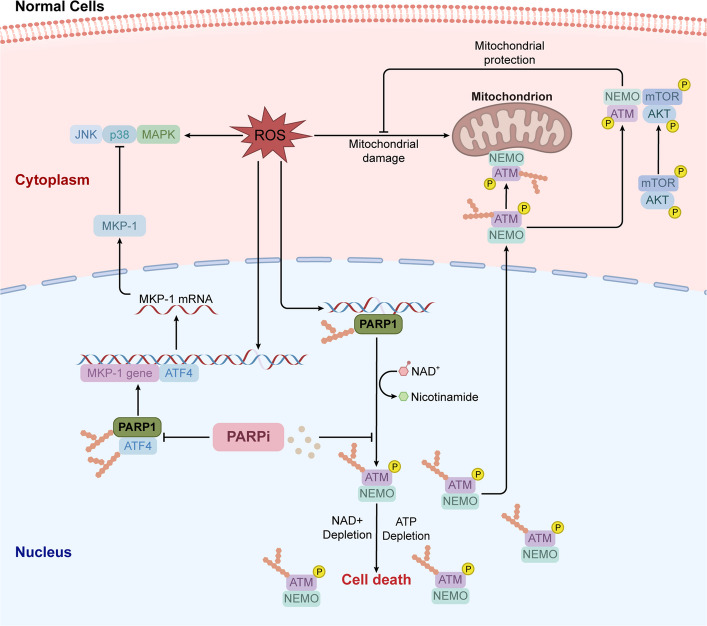
Mechanisms of action of PARPi in normal cells. In normal cells, PARPi inhibit DSB repair, leading to cell death, and also inhibit the ATM-NEMO complex activation and translocation, whereby mitochondria will continue to be induced and damaged by ROS. At the same time, PARPi can block excessive PARP activation, NAD^+^ depletion, and inactivation of JNK and p38 MAPK

Normal cells typically rely on HRR to repair DSBs. However, if the HRR function is impaired, PARPi may lead to genomic instability and cell death [206, 207]. Furthermore, PARPi can block excessive PARP activation, thereby preventing NAD^+^ depletion [208–210], and mitigating inflammatory cytokine storms (Fig. 5) [209, 211, 212]. Additionally, PARPi interfere with a mitochondrial protective pathway. PARP can activate the nuclear ATM-NEMO complex, which then translocates to the cytoplasm and attaches to the outer mitochondrial membrane. Akt is activated and protects mitochondria from reactive oxygen species (ROS)-induced damage, allowing cells to survive (Fig. 5) [213, 214]. PARPi inhibit this process and mitochondria then continue to be induced and damaged by ROS. At the same time, PARPi leave ATF4 unPARylated and, thus, able to bind to the promoter of the MKP-1 encoding DNA region. The resulting transcription of MKP-1 mRNA followed by translation of the MKP-1 protein leads to the inactivation of JNK and p38 MAPKs (Fig. 5) [215]. Collectively, these mechanisms suggest that the role of PARPi in normal cells is a double-edged sword. Therefore, it is necessary to weigh their efficacy and side effects in specific clinical applications.

### Mechanisms of PARPi in tumor cells

**Fig. 6:**
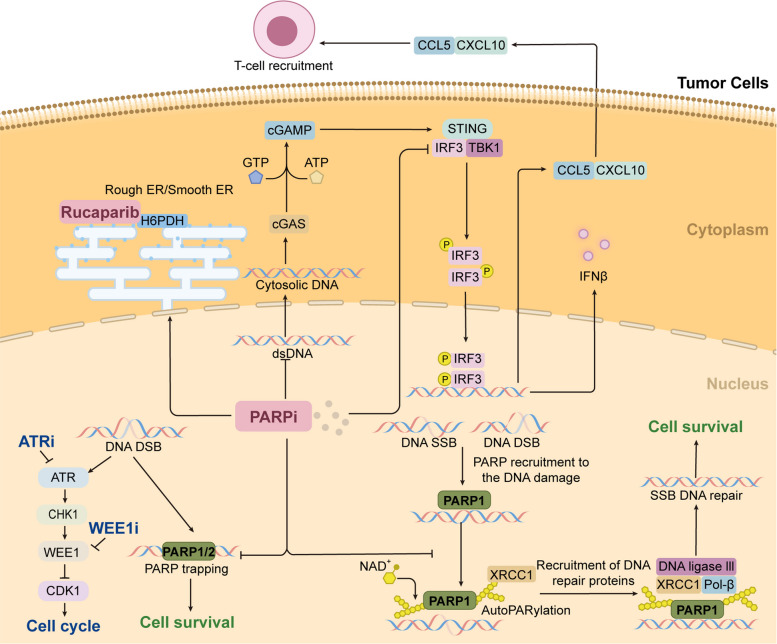
Mechanisms of action of PARPi in tumor cells. In tumor cells, PARPi inhibit PARP to modify itself and other proteins through PARylation, inhibit its recruitment of repair factors (XRCC1 DNA ligase III and Pol-β), and inhibit DNA SSBs and DSBs repair. PARPi inhibit PARP trapping, where PARP1/2 remain stably bound to DNA as toxic PARP–DNA complexes. PARPi activate the cGAS/STING signaling pathway, promotes the transcription of IFNβ and the secretion of CXCL10 and CCL5, recruiting CD8^+^ T cells, and enhancing the immune response in the tumor microenvironment. Meanwhile, PARPi promote type I interferon signaling through TBK1–IRF3 activation, enhancing anti-tumor immunity. Potential impact of PARPi on endoplasmic reticulum function may involve functional interplay with H6PDH. In addition, both WEE1i and ATRi can cooperate with PARPi to enhance DNA damage effects and further inhibit cell cycle progression

In tumor cells, when SSBs occur, PARP will bind to the DNA damage site and use NAD^+^ to generate PAR chains, recruiting DNA repair effector proteins, such as XRCC1, to complete the repair of SSBs (Fig. 6). PARPi will prevent the PARylation process by inhibiting the catalytic activity of PARPs, resulting in the incomplete repair of SSBs and DSBs and leading to cell death [216–219]. Beyond catalytic inhibition, accumulating evidence demonstrates that PARP inhibitors also act through PARP trapping, whereby PARP1/2 are stably retained on DNA as toxic PARP–DNA complexes [119]. The formation of these stabilized complexes induces replication fork stalling and subsequent collapse, thereby aborting replication fork restart. This effect is particularly pronounced in HR-deficient backgrounds, where compromised fork protection renders stalled forks vulnerable to Mre11-mediated degradation of nascent DNA [140]. By concurrently obstructing DNA repair and exacerbating replication stress, PARP inhibitors achieve potent and tumor-selective lethality.

In addition, PARPi lead to the accumulation of DNA damage, thereby activating intracellular cGAS, which catalyzes the production of cyclic GMP-AMP (cGAMP). cGAMP binds to and activates STING. The activation of STING further activates TANK-binding kinase 1 (TBK1), which then phosphorylates IRF3, allowing IRF3 to dimerize and transfer into the nucleus and promote the transcription of IFN-β (Fig. 6). The PARP7 inhibitor (RBN-2397) restores type I interferon signaling in tumor cells via the TBK1-IRF3 pathway, enhancing antitumor immunity and leading to tumor regression [101]. Meanwhile, PARPi activate the STING/TBK1/IRF3 pathway, resulting in increased expression of chemokines, such as the potent chemoattractants CXCL10 and CCL5, which target CD8^+^ T cells and enhance the immune response in the tumor microenvironment (Fig. 6) [220, 221]. In addition to directly inhibiting PARPs, PARPi may also affect cellular metabolism, thereby playing a broader role in cancer treatment [222]. For example, rucaparib is capable of binding to the NADP^+^-dependent hexose-6-phosphate dehydrogenase (H6PDH) in the endoplasmic reticulum, which is closely associated with redox regulation of this organelle and glucocorticoid metabolism [223]. Furthermore, PARPi can act synergistically with ATR and WEE1 inhibitors to suppress tumor progression. PARPi increase replication stress and promote the accumulation of DNA lesions, whereas ATR inhibition disables the S-phase cell cycle checkpoint and replication-fork protection, ultimately driving catastrophic fork collapse [224]. Critically, the synergy with WEE1 inhibition is a direct consequence of its location downstream of ATR. WEE1 blockade therefore bypasses the upstream ATR/CHK1 signal and forces a dysregulated cell cycle state [225]. This results in CDK1 hyperactivation, which drives excessive origin firing, nucleotide depletion, and premature mitotic entry. These events catastrophically amplify the replication stress and DNA damage initiated by PARPi [226]. These findings highlight that PARPi not only disrupt DNA repair and activate innate immune signaling but also modulate replication stress responses and cellular metabolism, thereby broadening their therapeutic potential in cancer treatment.

## Clinical applications of PARPi

### Clinical applications of PARPi as anti-tumor agents

In ovarian carcinoma, PARPi have redefined the standard of care by serving as maintenance therapy following platinum-based chemotherapy (Table 2). Olaparib was the first to demonstrate significant progression-free survival (PFS) benefit in BRCA-mutated patients [244]. For patients with newly-diagnosed advanced ovarian cancer (OC) and HR deficiency (HRD)-positive tumors, the combination of olaparib and bevacizumab has demonstrable efficacy [245, 246]. Niraparib has extended benefits in advanced ovarian cancer, HRD-positive and even HR-proficient populations due to its potent PARP trapping mechanism [119, 235, 236]. Rucaparib, with additional PARP3 inhibition, has also shown efficacy in platinum-sensitive recurrent ovarian cancer [247]. In breast cancer, olaparib and talazoparib are approved for germline BRCA-mutated, HER2-negative advanced disease, with talazoparib demonstrating superior PARP trapping and more robust tumor regression responses than olaparib [248]. Currently veliparib plus carboplatin is mainly employed to inhibit BRCA mutation-related breast cancer and triple-negative breast cancer [202].

**Table 2:** Generational comparison of PARPi in anti-tumor therapy

Generation	PARPi	Mechanisms	Approved/Investigated Indications	Clinical trial number	Common Adverse Effect	References
First-generation	Olaparib	PARP1/2 inhibition, moderate PARP trapping, synthetic lethality in HR-deficient cells	**Approved in Food and Drug Administration (FDA), European Medicines Agency (EMA) and China's National Medical Products Administration (NMPA):** BRCA-mutated ovarian cancer, HER2 negative breast cancer, pancreatic, and prostate cancers, peritoneal cancer, endometrial cancer; **Phase III trials:** Colorectal cancer, non-small cell lung cancer, small cell lung cancer, squamous cell cancer; **Phase II trials:** Bladder cancer, cervical cancer, gastric cancer, glioblastoma, head and neck cancer, HER2 positive breast cancer, osteosarcoma	NCT03402841; NCT03286842; NCT02184195; NCT02032823, NCT03278717, NCT03106987,NCT02392676, NCT05171816, NCT03534453, NCT01924533, NCT01844986, NCT02000622, NCT06712472, NCT03976323, NCT03740165, NCT04456699,NCT04624204,NCT05432791, etc	Nausea, fatigue, anemia	[227–230]
	Rucaparib	PARP1/2/3 inhibition, blocks DNA repair and induces cytotoxicity in HR-deficient cells	**Approved in FDA, EMA and NMPA:** BRCA-mutated ovarian cancer and BRCA-mutated metastatic castration-resistant prostate cancer; **Phase II trials:** Breast cancer, cervical cancer, endometrial cancer, gastric cancer, malignant-mesothelioma, pancreatic cancer, small cell lung cancer, solid tumors, triple negative breast cancer, urogenital cancer; **Phase I/II trials:** Non-small cell lung cancer	NCT02855944, NCT04227522, NCT01968213, NCT02975934, NCT03522246, NCT04455750, etc	Fatigue, nausea, anemia, Alanine Aminotransferase (ALT)/Aspartate Aminotransferase (AST) elevation, dysgeusia	[227, 230–234]
	Niraparib	Potent PARP trapping, PARP1/2 inhibition, active in some HR-proficient tumors	**Approved in FDA, EMA and NMPA:** Advanced ovarian cancer, fallopian tube cancer, peritoneal cancer; **Phase III trials:** Breast cancer, HER2 negative breast cancer, non-small cell lung cancer, prostate cancer, small cell lung cancer; **Phase II trials:** Cervical cancer, cholangiocarcinoma, CNS cancer, endometrial cancer, glioblastoma; mesothelioma; neuroendocrine tumors, pancreatic cancer; renal cell carcinoma, solid tumors, squamous cell cancer, triple negative breast cancer, urogenital cancer, uveal melanoma	NCT01847274, NCT06388733, NCT04915755, NCT03651206, NCT02655016, NCT04475939, NCT01905592, NCT04497844, NCT03602859, NCT03981796, NCT03748641, NCT06915025, NCT05615818, NCT03516084, etc	Thrombocytopenia, anemia, neutropenia, fatigue, hypertension	[230, 235–238]
	Talazoparib	Strongest PARP trapping, replication fork collapse and apoptosis	**Approved in FDA, EMA and NMPA:** BRCA-mutated breast cancer, HRR Gene–Altered metastatic castration-resistant prostate cancer (mCRPC); **Phase III trials:** Ovarian cancer; **Phase II trials:** Endometrial cancer, fallopian tube cancer, peritoneal cancer, solid tumors, triple negative breast cancer	NCT01945775, NCT03395197, NCT04821622, NCT03642132, etc	Anemia, neutropenia, thrombocytopenia, alopecia	[77, 227, 230, 239–241]
	Veliparib	PARP1/2 inhibition with mininal PARP trapping, sensitizes cells to DNA-damaging agents	**Phase III trials:** Fallopian tube cancer, HER2 negative breast cancer, non-small cell lung cancer, ovarian cancer, peritoneal cancer, triple negative breast cancer, Combination with carboplatin in triple-negative breast cancer (TNBC), non-small cell lung cancer (NSCLC); **Phase II/III trials:** Glioblastoma; **Phase II trials:** Brain metastases, breast cancer, colorectal cancer, germ cell and embryonal neoplasms, germ cell cancer, malignant melanoma, pancreatic cancer, rectal cancer, solid tumors	NCT02163694, NCT02470585, NCT02106546, NCT02264990, NCT02032277, NCT02152982, etc	Myelosuppression (notable with combination), nausea, fatigue	[202, 230, 242]
Next-generation	Pamiparib	High PARP1/2 selectivity, blood–brain barrier penetration	**Approved in NMPA:** BRCA-mutated recurrent ovarian cancer, fallopian tube cancer, peritoneal cancer; **Phase II trials:** Gastric cancer, HER2 negative breast cancer; **Phase I/II trials:** Glioblastoma, glioma, solid tumors	NCT03519230, NCT04164199, etc	Anemia, leukopenia, generally well tolerated in early studies	[204, 230]
	Fuzuloparib	PARP1/2 selectivity	**Approved in NMPA:** Platinum-sensitive recurrent ovarian cancer, fallopian tube cancer, peritoneal cancer; **Phase III trials:** Pancreatic cancer, prostate cancer; **Phase II trials:** Glioblastoma	NCT04691804, NCT06188455, NCT06533384, etc	Anemia, leukopenia, thrombocytopenia	[203, 230]
	Senaparib	Highly selective PARP1/2 inhibition with reduced off-target toxicity	**Approved in NMPA:** First-line maintenance in advanced ovarian cancer; **Phase II trials:** Small cell lung cancer, solid tumors; **Phase I/II trials:** Prostate cancer	NCT04822961, NCT07120451, NCT06617923, NCT04434482, NCT04089189, NCT05269316, etc	Nausea, fatigue, anemia, leukopenia	[205, 230, 243]

In prostate cancer, PARPi have demonstrated efficacy in metastatic, castration-resistant prostate cancer (mCRPC) harboring BRCA1/2 or other HR response gene alterations (Table 2). Olaparib gained approval following the PROfound trial [249], while combination strategies such as niraparib plus abiraterone have emerged to overcome resistance to androgen receptor signaling inhibitors [250]. In pancreatic cancer, a disease currently with limited treatment options, olaparib has shown benefit as maintenance therapy in germline BRCA-mutated patients [251], exploiting the same HR response vulnerabilities.

Recently developed agents, such as pamiparib, have gained approval for BRCA-mutated recurrent advanced ovarian, fallopian tube or primary peritoneal cancer [204]. Fuzuloparib has also been approved for platinum-sensitive ovarian cancers [203] (Table 2). Pamiparib offers high PARP1/2 selectivity and good blood–brain barrier permeability. Senaparib [205, 243], approved in 2025 as first-line maintenance for advanced ovarian cancer, represents a new generation of PARPi with reduced hematologic toxicity and improved tissue selectivity.

Collectively, PARPi have established themselves as a cornerstone of targeted therapy in precision oncology, driving the evolution of biomarker-driven, personalized treatment strategies and offering hope for durable disease control in genomically unstable tumors (Table 2).

### Applications of PARPi in antiviral therapy

Beyond oncology, PARPi have also emerged as a promising therapeutic strategy for antiviral treatment, targeting both DNA and RNA viruses (Table 3), exemplified by HBV and coronaviruses [252, 253], respectively, and other clinically-significant viruses, such as high-risk human papillomavirus (HPV)-associated malignancies in the endocervix, head, and neck [263, 264] and pathogens with pandemic potential, including IAV [259]. Recent studies have demonstrated that the combination of PARPi with CRISPR/Cas9 technology can significantly enhance the antiviral efficacy against HBV by directly targeting covalently closed circular DNA (cccDNA). For instance, the inhibition of NHEJ pathways through PARPi has been shown to augment the antiviral effects of HBV-CRISPR, leading to marked reductions in both cccDNA and pregenomic RNA levels in infected hepatocytes [252]. Furthermore, stenoparib has demonstrated dose-dependent antiviral activity against SARS-CoV-2 and the seasonal human alphacoronavirus HCoV-NL63, through a dual mechanism of action by interfering with both viral entry and replication processes [253]. Additionally, the combination of PARPi with favipiravir for the treatment of SARS-CoV-2 has demonstrated potential synergistic effects that could enhance the overall treatment efficacy [254, 255]. Olaparib ameliorates IAV-induced pneumonia in mice by inhibiting the PARP1 activity, modulating inflammatory cytokines, reducing leukocyte infiltration, and inhibiting the NF-κB signaling pathway [259]. In HPV-associated tumors, such as cervical cancer and head and neck squamous cell carcinoma (HNSCC), persistent virus infection leads to damage to the host's DNA repair mechanisms, making tumor cells particularly sensitive to PARPi. Preclinical and early clinical studies have shown that PARPi exert antitumor effects also by enhancing the sensitivity of DNA damage treatments, such as radiotherapy and cisplatin, thereby improving treatment response rates and delaying tumor progression [264–267]. The clinical application of PARPi in other viral infections, including KSHV [258], and HIV [256], is also underway, with preliminary data suggesting that they may inhibit viral replication effectively and potentially heralding the development of broad-spectrum antivirals.

**Table 3:** Clinically significant viruses and PARPi applications

Virus	PARPi	Mechanism of Action	Experimental Model/Stage	Common Adverse Effect	Reference
HBV	Olaparib	Inhibits NHEJ, enhancing CRISPR-mediated cccDNA cleavage	HepG2-hNTCP-C4 cells, primary human hepatocytes (PHHs)/Preclinical	No human safety data	[252]
SARS-CoV-2/HCoV-NL63 Human Coronaviruses	Stenoparib, CVL218 (mefuparib)	Dual inhibition of viral entry and replication, synergistic with remdesivir or favipiravir	Vero E6, Calu-3 cells/Preclinical	No human safety data	[253–255]
HIV	Talazoparib, 3-aminobenzamide, Olaparib, Talazoparib, 5-aminoisoquinolinone, PJ34	Enhances NK cell-mediated cytotoxicity, combination with the HDAC inhibitor vorinostat, inhibits HIV-1 LTR activation through NFκB suppression, disrupts actin cytoskeleton rearrangements via Rho GTPase inhibition	J-Lat cell lines, human monocyte-derived macrophages (MDM)/Preclinical	No human safety data	[256, 257]
KSHV	AZD2461	Induces DNA damage, reduces cell proliferation, combination with the CHK1 inhibitor UCN-01	Latency models (PEL cells)/Preclinical	No human safety data	[258]
IAV	Olaparib	Modulating inflammatory cytokines, reducing leukocyte infiltration, and suppressing the NF-κB signaling pathway	Mice model	No human safety data	[259]
HPV/HBV related cancers	Cervical cancer: Veliparib, Olaparib; HNSCC: Olaparib, Niraparib, Veliparib, Talazoparib, Rucaparib, CEP-9722	Cervical cancer: Enhance DNA damage, combination with the ATR inhibitor AZD6738; HNSCC: Enhance DNA damage, boost immune responses by inducing PD-L1 expression, combination with cisplatin	Cervical cancer: Phase I/II trials; HNSCC: Phase I-III trials	Anemia, neutropenia, thrombocytopenia, dyspnea, leukopenia	[260–262]

Notably, viruses from the class *Bunyaviricetes*, such as Crimean–Congo hemorrhagic fever virus (CCHFV) [268] and Rift Valley fever virus (RVFV) [269], have been shown to influence PARP cleavage during infection. While no direct studies have yet tested PARPi against bunyaviruses, these observations highlight the involvement of PARPs in bunyavirus-host interactions. Thus, bunyaviruses may serve as illustrative examples pointing to the broader possibility that PARPi could have therapeutic relevance in additional viral contexts beyond those currently reported.

### Applications of PARPi in other diseases

It is increasingly evident that PARPi offer therapeutic potential beyond oncology and viral infections, particularly in non-malignant diseases driven by DNA damage, oxidative stress, and inflammatory signaling.

In neurodegenerative diseases, particularly Parkinson's disease, PJ34, a selective PARP1 inhibitor, has shown significant neuroprotective effects in experimental traumatic brain injury (TBI) by reducing neuronal death and microglial activation [26]. The PARP inhibitors 3-aminobenzamide (3-AB) or 5-aminoisoquinolinone (5-AIQ) reduce excessive PAR formation in the process of DNA damage repair by inhibiting the activity of PARP enzymes, thus alleviating the inflammatory response, apoptosis, and tissue damage, and, thus, have potential therapeutic effects in varied clinical entities, such as spinal cord injuries and stroke [27]. PARP14 inhibitors (such as RBN-3143) have shown therapeutic potential for atopic dermatitis (AD) in preclinical studies and can reduce the level of inflammatory factors, including Th17 cytokines [28]. In cardiovascular diseases, PARPi, such as 3-AB, PJ-34, INO-1001, BGP-15, and GPI 6150, reduce the consumption of NAD^+^ and ATP by inhibiting the overactivation of PARP1, thereby alleviating oxidative stress and inflammatory responses and protecting cells from necrosis. Considering heart failure and myocardial hypertrophy, PJ-34, INO-1001, and L-2286 play a role by improving contractile function, reducing myocardial cell death, and decreasing myocardial hypertrophy and fibrosis. In circulatory failure, 3-AB, PJ-34, and INO-1001 improve survival by promoting myocardial contractility and vascular function, reducing inflammation and tissue damage. In diseases such as hypertension, cardiovascular aging, atherosclerosis and vascular remodeling, diabetic cardiovascular complications, and angiogenesis, PJ-34, INO-1001, and other inhibitors have shown therapeutic potential by improving endothelial function, reducing plaque size and promoting plaque stability, reducing endothelial dysfunction, improving contraction and vascular function, and reducing VEGF or bFGF-induced angiogenesis [29]. Lastly, in organ fibrosis, PARPi, particularly HYDAMTIQ, inhibit TGF-β signaling, reduce fibroblast activation, and attenuate lung fibrosis progression in mice [30]. Olaparib inhibits the development of liver fibrosis by inhibiting PARP1 activity, reducing inflammatory responses, apoptosis, activation of TGF-β/Smad signaling pathway and oxidative stress [30].

In inflammatory and autoimmune diseases, olaparib effectively reduces the number of monocytes in the blood of mice and exerts anti-inflammatory effects by increasing IL-10 levels and inhibiting the concentrations of IL-1β and IL-6 in Crohn's disease models. 3-AB improves rectal bleeding, reduces blood glucose levels, lowers serum IL-1β levels, decreases weight loss, and improves histological scores of colon tissue sections in a mouse model of colitis-related diabetes [270]. In addition, RBN-3143, as a PARP14 inhibitor, is being used in clinical trials to treat atopic dermatitis, which primarily relieves symptoms in patients with atopic dermatitis by controlling inflammation [271].

In metabolic disorders, olaparib can prevent and alleviate NAFLD and non-alcoholic steatohepatitis (NASH), and diminish liver lipid accumulation by regulating liver lipid metabolism-related signaling pathways. PJ34 attenuates hepatocyte lipid accumulation in a mouse model of NAFLD induced by a high-fat and high-sucrose diet. TIQ-A reduces plasma cholesterol levels and reduces plaque formation in high-cholesterol diet-induced atherosclerotic mice. G-007LK reduces serum triglycerides, non-esterified fatty acids and total cholesterol levels, and improves obesity-related metabolic disorders in db/db mice. INO-1001 reduces LDL-C levels, inhibits the expression of inflammatory factors, and reduces the development of atherosclerosis in a murine model [102].

Although no PARPi are currently clinically approved outside oncology, the growing body of mechanistic and preclinical evidence underscores the promising scope of PARPi repurposing in a wide range of chronic and degenerative diseases.

### PARPi mediated resistance to clinical therapy

PARPi are increasingly recognized as a dynamic process that evolves with disease progression and treatment duration [217, 272]. In ovarian cancer, continuous circulating tumor DNA (ctDNA) monitoring found that approximately 28% of the patients late in treatment developed acquired mutations in HRR-related genes, resulting in a significant reduction in PARPi sensitivity [273]. Meanwhile, drug resistance to PARPi can also be generated through other mechanisms, such as KAT6A-mediated liquid–liquid phase separation (LLPS), which reduces the retention of PARP1 at DNA damage sites and where the inhibition of KAT6A partially restores sensitivity [274]. Similarly, the efficiency of PARP1 trapping at DNA lesions, a key mechanism of PARPi cytotoxicity, is itself regulated by time-dependent, drug–target binding kinetics. Variations in the dissociation rate constant influences the persistence of PARP1–drug complexes and ultimately the depth of DNA repair inhibition [275]. It is worth noting that temporal changes in drug resistance are also seen in virus-associated cancers. In EBV-positive gastric cancer, EBNA1 enhances the sensitivity to olaparib by inhibiting the ATR kinase activity, however, may lead to drug resistance through the p38 MAPK pathway activation or ATR compensatory activation after long-term treatment [276]. Moreover, the heterogeneity within CDK12-mutated prostate cancers demonstrates that distinct mutational patterns drive differential therapeutic outcomes, with truncating mutations sustaining PARPi sensitivity and kinase-domain mutations leading to an intermediate reactive drug resistance [277]. Together, these studies reveal that genomic evolution, drug binding dynamics, and chromatin remodeling all contribute to temporally-regulated resistance, and highlight the stage specificity of drug resistance mechanisms, and the need for timely adjustment of treatment strategies by dynamic monitoring.

Given these temporally and contextually-regulated resistance mechanisms, monotherapy with PARPi is insufficient to ensure durable clinical responses [278]. Instead, combinatorial strategies appear essential to counteract both genomic adaptations and microenvironmental suppression [200, 279]. Preclinical studies have shown that STING agonists can reprogram M2-like TAMs toward antitumor M1 phenotypes and synergize with PARPi to restore immune activation [280], while immune checkpoint blockade targeting PD-1/PD-L1 can resensitize resistant tumors to T cell killing [281]. Rational drug combinations with DDR-targeting agents, such as ATR or WEE1 inhibitors, may further prevent the rapid outgrowth of resistant clones in genetically-heterogeneous tumors [282]. For moderately sensitive patients, such as those harboring CDK12 kinase domain mutations, the combination strategy of PARPi and other synergistic drugs can be considered in the early stage of treatment to avoid rapid screening of drug-resistant clones under the pressure of single agents. For sensitive subtypes, such as truncation mutations, CDK12 mutation status and replication stress-related biomarkers can be dynamically monitored to adjust treatment regimens early in disease progression [277].

In summary, these findings indicate that the most promising strategy to circumvent PARPi resistance is the application of stage-tailored and context-specific combination therapies, underpinned by dynamic biomarker monitoring.

### Potential side effects and safety concerns of PARPi

Although PARPi are generally well tolerated, their toxicity profiles are not uniform and are strongly shaped by the cell- and tissue-specific expression of PARP enzymes, drug-specific pharmacodynamics, and organ-level pharmacokinetics. Hematologic toxicities, including anemia, neutropenia, and thrombocytopenia, represent the most common adverse events and reflect the high sensitivity of the bone marrow compartment [9, 283]. Mechanistically, PARP1 is broadly expressed, with particularly high abundance in lymphoid and hematopoietic cells, and experimental models demonstrate that combined PARP1/2 deficiency disrupts B cell development [284]. This underpins the clinical observation that hematological toxicity is a hallmark adverse effect of PARPi treatment. Furthermore, the severity of hematologic adverse events correlates with the extent of PARP trapping (rather than catalytic inhibition), with talazoparib exhibiting the strongest trapping activity and the highest hematologic toxicity [119, 248]. Clinical trials of niraparib additionally reveal a host factor–dependent pattern: patients with lower body weight or reduced baseline platelet counts experience a higher incidence of thrombocytopenia. As a result, individualized starting doses based on weight and platelet counts have become a standard practice to mitigate severe hematologic events [285, 286]. Rare, but clinically significant, late-onset complications include myelodysplastic syndrome (MDS) and acute myeloid leukemia (AML), which occur at a cumulative incidence of ~ 0.7%, typically after prolonged exposure, and are thought to represent organ-specific (hematopoietic) toxicity [287].

Beyond the bone marrow, other organ systems exhibit distinct toxicity signatures. In the gastrointestinal tract, PARP1 plays a role in epithelial injury responses, and preclinical data suggest that PARPi protected intestinal barrier integrity [288]. Clinically, nausea and vomiting are the most frequently reported gastrointestinal adverse events, whereas diarrhea and constipation are less common [289]. In the cardiovascular system, niraparib uniquely inhibits norepinephrine and dopamine transporters, leading to hypertension and tachycardia, which necessitates close blood pressure monitoring during the early treatment phase [290]. In the liver, rucaparib is frequently associated with transient elevations of alanine aminotransferase (ALT)/aspartate aminotransferase (AST), typically within the first few cycles and without concurrent bilirubin elevation, indicating a largely reversible hepatocellular reaction [291]. Renal toxicities also show organ-specific patterns: olaparib inhibits proximal tubular transporters (OCT2 and MATE1/2-K), resulting in a benign increase in serum creatinine without true glomerular filtration rate (GFR) impairment. In such cases, cystatin-C is recommended for more accurate assessment of renal function [292, 293].

Emerging strategies to reduce cell- and tissue-specific toxicity include the development of selective PARP1 inhibition (e.g., saruparib). Preliminary data from preclinical studies suggest that such selective inhibitors may have a reduced impact on hematological parameters [294].

In summary, the heterogeneity of PARP expression across cell types and organ systems plays a central role in shaping the adverse event spectrum of PARPi therapy. Clinical practice has increasingly shifted toward organ-specific monitoring and individualized dosing strategies, including weight- and platelet-adjusted initiation, early and frequent laboratory surveillance (liver function and renal biomarkers), and cardiovascular monitoring for agents, such as niraparib. Awareness of these cell- and tissue-specific determinants enhances the clinical relevance of PARPi safety management and informs the rational design of next-generation inhibitors with improved tolerability.

## Conclusions and prospects

In this review, we have provided a comprehensive overview of PARP biology, PARPi, and existing and potential clinical applications. We have introduced the mechanisms and the known biological functions of PARPs, outlining their classification, structural characteristics, and canonical functions in DNA repair, parthanatos, and cellular homeostasis, alongside their non-canonical roles in transcriptional regulation, immunity, and metabolism. We have outlined the roles of PARPs in disease pathogenesis, including their contribution to genomic instability, tumor cell death regulation, tumor microenvironment remodeling, and viral infections, involving both DNA and RNA viruses. Beyond cancer and viral biology, we also highlighted their functions in neurodegenerative, cardiovascular, fibrotic, autoimmune, inflammatory, and metabolic diseases. Finally, we have reviewed the mechanisms of action of PARPi in normal and tumor cells, their clinical applications in oncology, antiviral therapy, and other disease contexts, as well as the challenges of drug resistance, toxicity, and safety management. Collectively, these insights position PARPs as central molecular regulators and PARPi as versatile therapeutic agents with implications far beyond oncology.

### Next-generation PARPi and novel modalities

While current PARPi (e.g., olaparib, niraparib, rucaparib, and talazoparib) have transformed the treatment of HRR-deficient cancers, their broader application is limited by off-target toxicities and insufficient selectivity [295]. The next wave of innovation will involve highly selective PARPi [296], dual-target compounds, and novel modalities, such as PROTAC-based degraders [297] or allosteric inhibitors. These strategies may enhance efficacy, reduce adverse effects, and expand indications to patients without classical BRCA mutations. Furthermore, the potential of targeting other PARP family members (e.g., PARP7, PARP9, and PARP14) in immune and viral contexts remains underexplored and warrants systematic investigation; however, rigorous preclinical and translational studies are required to ensure that improved pharmacologic properties can be translated into sustained clinical benefit.

### Overcoming resistance: novel combination strategies

A major barrier to PARPi efficacy is the emergence of resistance, frequently through restoration of HRR, stabilization of replication forks, or drug efflux mechanisms [298–300]. Rational combination strategies—such as PARPi with ATR/CHK1 inhibitors [301], epigenetic modulators [302, 303], or immune checkpoint blockade [304]—offer promising avenues to resensitize tumors and potentiate antitumor immunity. The challenge lies in identifying optimal dosing regimens, scheduling, and biomarker-guided patient selection to balance efficacy with tolerability. Addressing these questions will be critical for sustaining long-term responses in the clinic.

### Biomarker development and personalized therapies

The clinical translation of PARPi is still constrained by the lack of robust and dynamic biomarkers. Current reliance on BRCA1/2 and HRR gene mutations fails to capture the full spectrum of responsive patients [305]. Future work should focus on integrating liquid biopsy-based markers (ctDNA, and exosomal RNA), functional HRR assays, and single-cell/spatial multiomics platforms to monitor treatment response and resistance evolution in real time. Embedding such diagnostics into clinical decision-making frameworks will enable a new era of precision medicine approaches and extend PARPi benefits beyond narrowly-defined patient populations.

### Beyond DNA repair: expanding therapeutic horizons

Although PARPs are traditionally recognized for their DNA repair roles, they also participate in immune signaling, transcriptional regulation, metabolism, and stress responses [306, 307]. These broader functions suggest therapeutic potential in non-oncologic contexts, including neurodegeneration, fibrosis, cardiovascular disorders, and autoimmune diseases [179, 186, 195, 196, 306–308]. The challenge is to delineate the isoform-specific and context-dependent roles of PARPs to avoid unintended consequences of long-term inhibition [309]. Moreover, immunomodulatory effects of PARPi could be leveraged in viral infections or combined with immunotherapies, thereby broadening their clinical reach [307].

Overall, while PARPi have already redefined cancer therapy, their full potential remains unrealized. Future efforts must resolve key gaps: (1) clarifying isoform-specific functions across disease contexts; (2) designing next-generation, safer PARPi; (3) establishing robust biomarkers for personalized treatment; (4) optimizing rational combination strategies; and (5) elucidating the shared mechanisms of PARPs in cancer, viral infections, and other diseases. Addressing these research questions will not only enhance therapeutic precision but also determine whether PARPi can evolve from oncology-focused agents into broad-spectrum modulators of human disease.

## Data Availability

No new data were generated or analyzed in this study. This work is a review study and does not rely on any external data.
